# Medical Students

**Published:** 1854-02

**Authors:** 


*Medical Students.*—An exchange speaks of an apparent falling off in the attendance at various Medical Colleges, during the present season. One College has gone down bodily; some of the N. York Schools have less than their usual number and the same is affirmed of some of the Cincinnati Colleges. Whether this diminution extend farther, we cannot say—but can only state for ourselves that the attendance at the Iowa Medical College is larger than at any previous year, and more than twice as large as that during the last session. We are greatly encouraged by so prominent a fact, after the depressing circumstances which have surrounded the Institution, but which have passed away and left it in a much more vigorous condition than ever before.

